# A Review on Multi-Scale Toughening and Regulating Methods for Modern Concrete: From Toughening Theory to Practical Engineering Application

**DOI:** 10.34133/research.0518

**Published:** 2024-12-26

**Authors:** Jinhui Tang, Chang Gao, Yi Li, Jie Xu, Jiale Huang, Disheng Xu, Zhangli Hu, Fangyu Han, Jiaping Liu

**Affiliations:** ^1^ School of Materials Science and Engineering, Jiangsu Key Laboratory of Construction Materials, Southeast University, Nanjing, China.; ^2^ State Key Laboratory of High Performance Civil Engineering Materials, Jiangsu Sobute New Materials Co. Ltd., Nanjing, China.

## Abstract

Concrete is the most widely used and highest-volume basic material in the word today. Enhancing its toughness, including tensile strength and deformation resistance, can boost the structural load-bearing capacity, minimize cracking, and decrease the amount of concrete and steel required in engineering projects. These advancements are crucial for the safety, durability, energy efficiency, and emission reduction of structural engineering. This paper systematically summarized the brittle characteristics of concrete and the various structural factors influencing its performance at multiple scales, including molecular, nano-micro, and meso-macro levels. It outlines the principles and impacts of concrete toughening and crack prevention from both internal and external perspectives, and discusses recent advancements and engineering applications of toughened concrete. In situ polymerization and fiber reinforcement are currently practical and highly efficient methods for enhancing concrete toughness. These techniques can boost the matrix’s flexural strength by 30% and double its fracture energy, achieving an ultimate tensile strength of up to 20 MPa and a tensile strain exceeding 0.6%. In the future, achieving breakthroughs in concrete toughening will probably rely heavily on the seamless integration and effective synergy of multi-scale toughening methods.

## Introduction

Concrete, a heterogeneous engineering material, is formed through the hydration and hardening process of sand and gravel aggregates, cement, water, as well as chemical and mineral admixtures, exhibiting a multi-phase, multi-scale nature characteristics [1,2]. Its usage has accounted for over 90% of the total volume of the primary construction building materials, with several advantages including the benefits of abundant raw materials, robust plasticity, and high compressive strength [3,4]. This material plays a pivotal role in facilitating the construction of significant projects in areas such as transportation, energy, and national defense. As the development of technology, higher-quality construction structures are urgently needed with remarkable advantage in special application requirements or extreme service environment. Therefore, the requirements for concrete materials with high strength, high crack resistance, and high durability are put forward. Nevertheless, due to the “bottom-up” quasi-brittle attributes of concrete materials, they tend to present poor toughness and ductility. Moreover, as the compressive strength rises, their brittle nature becomes increasingly pronounced, often resulting in easily cracking. Once concrete cracks, it not only accelerates the transmission rate of harmful media and aggravates the degradation of the material’s own performance and the corrosion of steel bars, greatly shortening the service life of the structure, but also reduces the bearing capacity, seriously affecting the structural safety under extreme loads [5–8]. Therefore, developing high-strength and high-crack-resistant concrete and achieving performance innovation are of great significance for improving the service life of structures and innovating high-performance sustainable structures.

Metals and polymer materials are widely recognized for their remarkable toughness. This is primarily attributed to their capacity to absorb energy through mechanisms such as atomic structure dislocation in metals and molecular chain configuration changes in polymers when subjected to external forces. It can help to impede crack initiation, minimize fracture risks, and ultimately enhance toughness. Therefore, enhancing resistance against crack initiation, propagation, and unstable growth serves is the crucial approach to achieve material toughening [9,10]. Then, the enhancement of toughness for concrete has emerged as a focal point of research, primarily achieved through the incorporation of nanomaterials, organic polymer materials, and fibers. The incorporation of nanomaterials and functional polymers contributes to cement hydration, reinforcing the hydration products such as element-doped calcium–silicate–hydrate (C-S-H) gel and ordered calcium hydroxide (CH) crystals at the nano- to microscale [11–14]. This incorporation decreases porosity, establishes a polymer network structure that alleviates internal stress, enhances the interface transition zone (ITZ), fortifies the matrix, reduces nano-microscale defects, minimizes the risk of crack initiation, and ultimately achieves internal toughening [15–17]. Meanwhile, the incorporation of fibers can impede the formation and non-steady-state expansion of micro-cracks based on the principle of physical bridging at a micro-macro scale, leading to strain hardening and multi-crack propagation in concrete [18,19]. This process significantly boosts the fracture energy of concrete, refines crack width, and ultimately accomplishes external toughening. Hence, the toughening of concrete materials can be attained through both internal and external methods. Generally, in recent years, a continuous stream of toughening methods for concrete materials has surfaced, with the aim of enhancing its crack resistance. These methods have gradually found application in major practical projects worldwide, not only achieving lighter concrete materials but also enhancing their long-term durability.

In this paper, first, we focus on the multi-scale brittle characteristics of concrete, followed by a detailed review of the theory behind the toughness exhibited by high-toughness materials in nature. Subsequently, the “Toughening Approaches and Principles of Concrete” section provides a summary of existing toughening techniques at multi-scales from the perspective of materials and fracture mechanics (cracks). Moving forward, the “Preparation and Applications of Toughened Concrete” section outlines the current state of preparation and application of toughened concrete based on the aforementioned toughening measures. Finally, in the “Prospective Work in Toughing Concrete” section, field applications related to multi-scale toughening of concrete are proposed, along with prospects for future advancements in this area.

## Theory of Toughness

### Brittleness of concrete

The brittleness of concrete refers to the sudden fracture without obvious plastic deformation when subjected to external forces, which is frequent to produce circular cracks and a large number of small cracks before reaching the design strength. Generally, with the increase of concrete strength grade, brittle fracture failure becomes more significant.

**Fig. 1.**
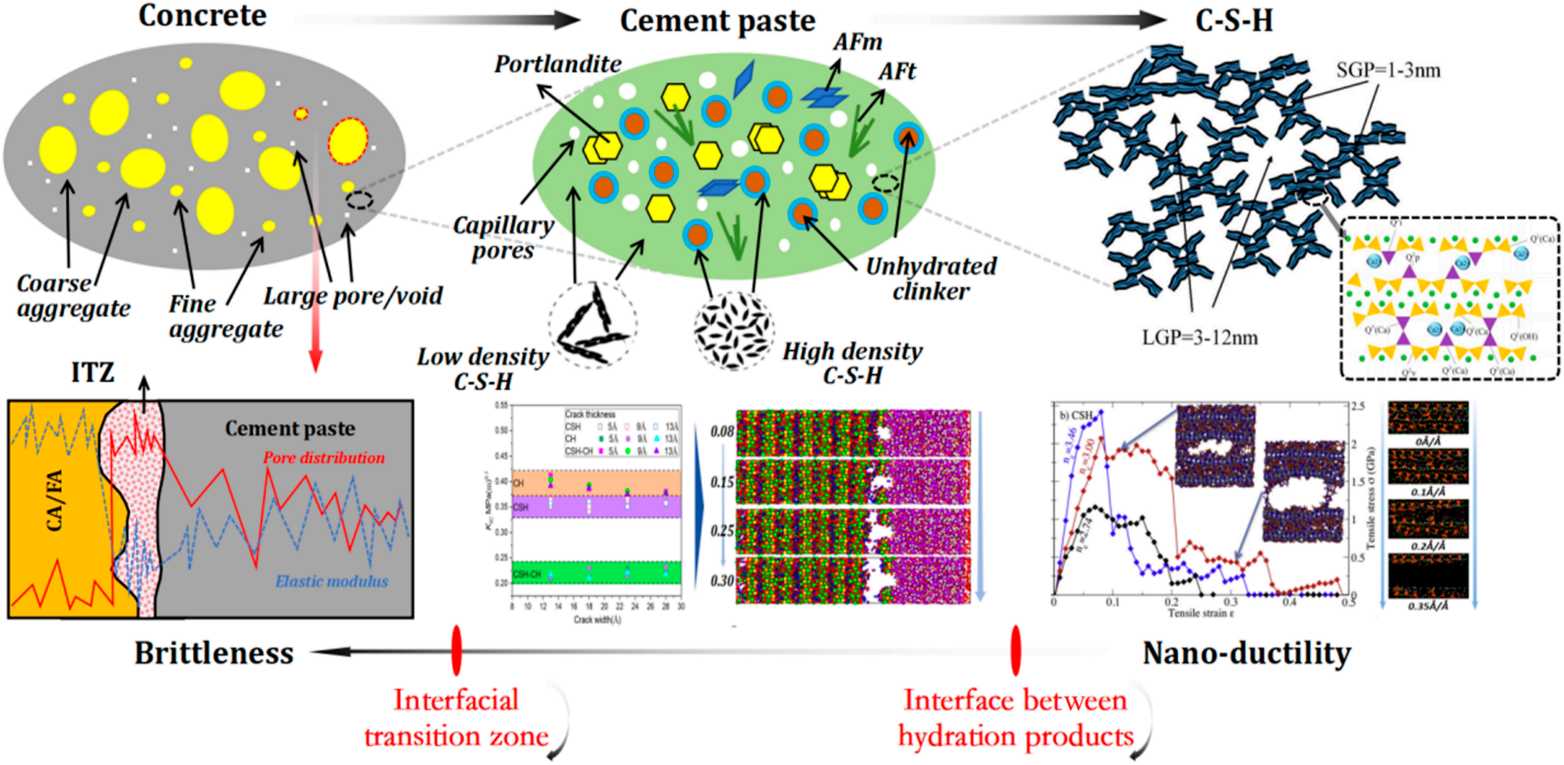
Multi-scale brittle characteristics of concrete [20–22].

C-S-H is the main amorphous hydration product of cement-based materials (60% to 70%), whose molecular structure consists of [Si–O] tetrahedron and [Ca–O] octahedron. The unique defective Tobermorite structure of C-S-H determines its “nanoductility” observed at the atomic scale. As for other hydration products (CH, ettringite, ...), directional covalent bonds and ionic bonds are the basic structural existence forms. Accordingly, no deformation systems inside offers plastic strain before fracture, which determines the inherent brittleness for these crystalline phases. However, as the scale increases, the cement hydration system is gradually becoming a complex multi-phase system with the coexistence of gas, liquid, and solid. The introduction of pore defects and product interfaces leads to significant issues of strength degradation and toughness loss [23]. When it comes to concrete, the addition of aggregates with different particle sizes further introduces the interfacial transition zones (ITZs), which can be regarded as the “weak zones” inside concrete due to higher porosity. Therefore, concrete generally exhibits sudden brittle fracture macroscopically during service life. The brittle nature of concrete originates from the barrier issues in the scale transition of mechanical properties [23], as shown in Fig. 1, which will be explained in detail in the “Toughening Approaches and Principles of Concrete” section.

### Theory of toughness

The fracture toughness of ductile materials in nature mainly comes from the suppression of crack initiation and propagation at the crack tip, as well as the connection and shielding behind the crack tip [24], which is closely related to the microstructure and the debonding or fracture process at the crack tip. The main manifestations include the improvement of crack initiation resistance (from the perspective of material), crack blunting, twisting of the crack propagation path, and bridging behind the crack tip (from the perspective of energy dissipation mechanism).

**Fig. 2.**
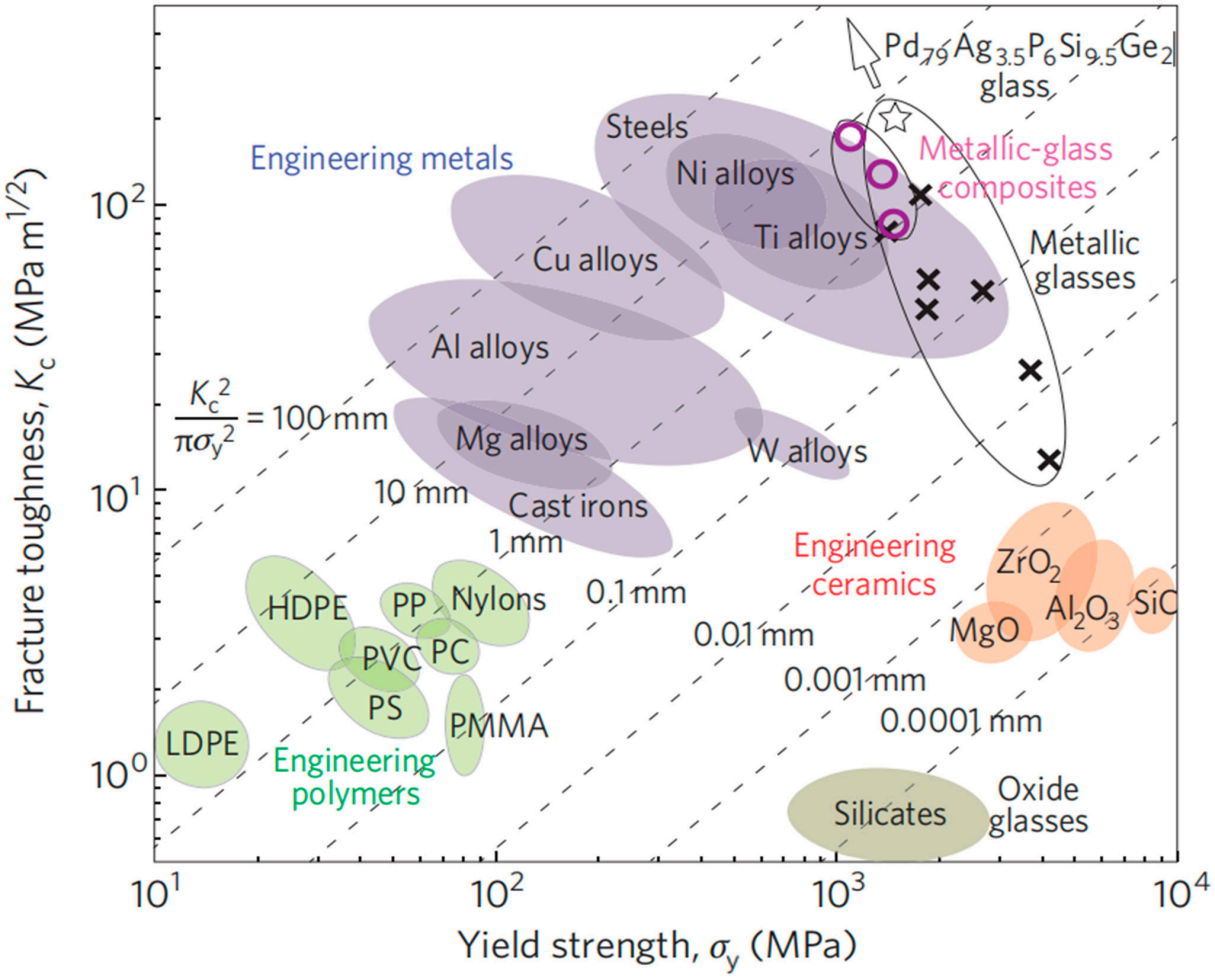
Ashby plot of the strength–toughness relationship for common materials [24].

From the perspective of the material, the crack initiation toughness, that is, the crack resistance of the material, is related to the size of plastic zone inside [24]. The larger the plastic zone size, the stronger the deformation ability, and the better the toughness, as shown in Fig. 2. Because of the flexibility of molecular chains and the weak nonpolar interactions between chains (van der Waals forces or hydrogen bonds), polymers can undergo plastic deformation and absorb energy when impacted through mutual sliding between molecular chains, preventing the rapid propagation of cracks. For most metals, due to their internal nonpolar metal bonds and lower barriers, electrons and metal atoms are allowed to undergo displacement under external forces rather than direct fracture. The dislocations (edge dislocations or screw dislocations) inside the lattices of metal provide a mechanism for plastic deformation.

In contrast, inorganic materials such as ceramics are often composed of directional covalent bonds, which are lack of independent slip systems and have difficult in releasing energy through plastic deformation. The plastic zone size is very small. In addition, due to the presence of point defects, line defects, surface defects, and micro-cracks/pores/impurities with various sizes, the cracks are prone to propagate along specific grain boundaries and suddenly fracture. Therefore, for brittle inorganic materials, the initial fracture toughness can be improved by expanding its own plastic zone size or introducing other materials with larger plastic zone. Obviously, the latter is easier to implement, such as the preparation of organic–inorganic [25]/organic–metallic composites [26].

From the perspective of cracks, the mechanism of crack blunting and twisting of the crack propagation path can be illustrated by using the concept of “sacrificial bonds”. Taking hydrogels as an example, a kind of typical high-toughness material, its toughness comes from the fracture of a large number of sacrificial bonds (weak or overstressed bonds) during the fracture process [27]. The sacrificial bonds preferentially fracture, while the rest of the network remains intact, which can significantly reduce the stress concentration in the strong bond network caused by the crack tip. The observed crack blunting can efficiently avoid local damage and enhance the fracture energy of hydrogels. This toughening mechanism by optimizing the combination of strong bonds and weak bonds provides abundant design ideas for various materials. For hydrophobic multiple-network elastomers, the priority fracture of sacrificial bonds can also increase the area of the debonding zone at the crack tip of the material to avoid brittle fracture [28].

The cracking bridging is principally achieved by the wake of the crack, and it encompasses grain bridging [24,29–31], polymer bridging [15,32], and fiber bridging. The material can be strengthened and toughened through multi-scale crack bridging. For instance, the plate-shaped grain network structure formed by hot pressing of silicon carbide (SiC) ceramics [31] can isolate the crack tip from the external load through grain bridging and pull-out mechanism when micro-cracks occur, thereby hindering crack propagation. Notably, the grain bridging primarily occurs in the presence of grain-boundary films at the nanoscale, allowing cracks to deflect or bend around the grains, thus accelerating the energy dissipation at the tip and preventing brittle failure. From millimeters down to hundreds of nanometers, the nacre structure [33] can interlock initially through mineral bridging, generating frictional resistance during the local sliding at the interface. Additionally, at the lower scale of tens of nanometers, organic material bridging between nanoparticles can promote the rotation and deformation of nano-particles, and enhance the damage tolerance of nacre. Human bones [24] are another exemplary material that amalgamates strength and toughness. The crack bridging facilitated by the mineralized collagen fibrils can effectively control crack propagation and enhance the toughness.

**Fig. 3.**
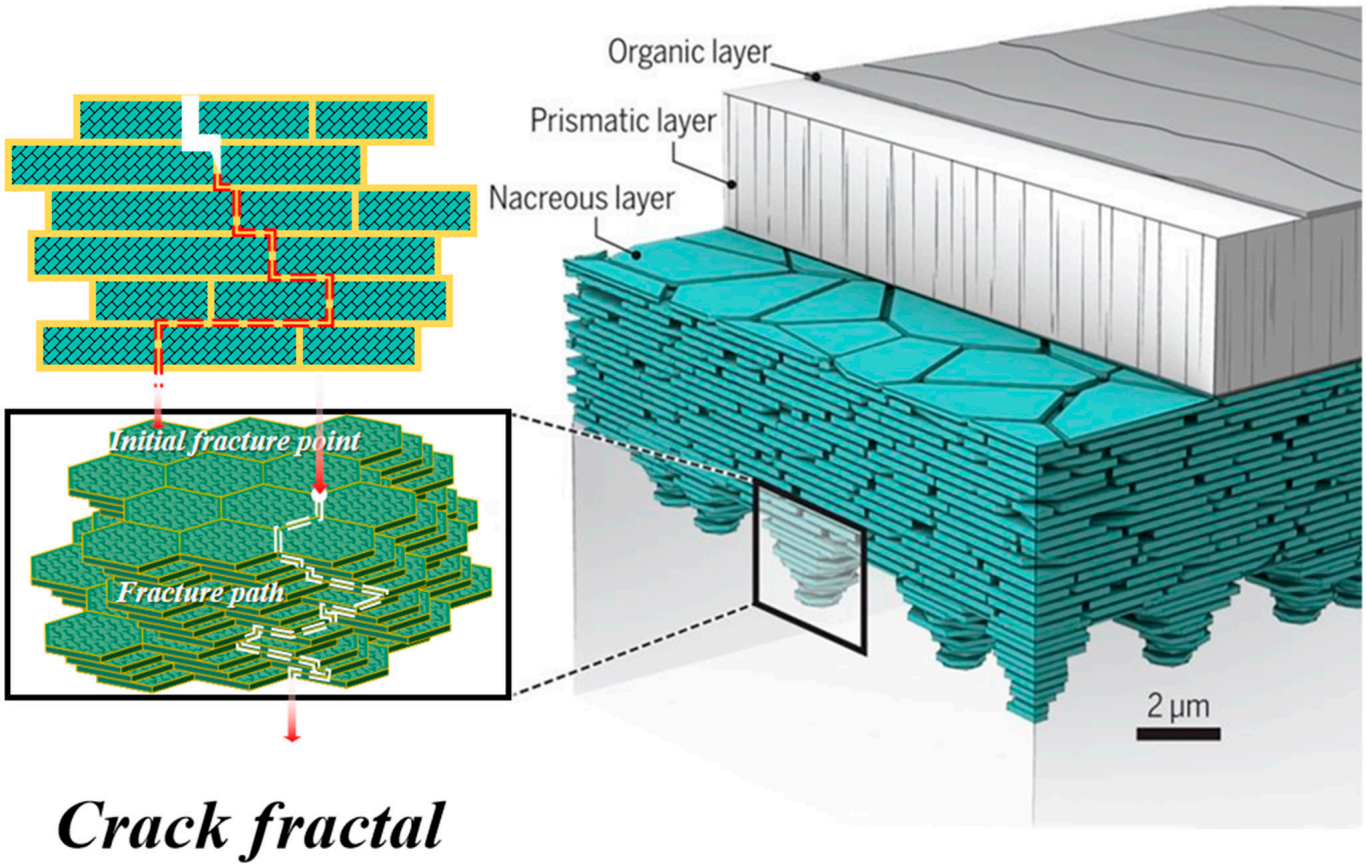
Multi-scale toughening mechanism of natural nacre [40].

Accordingly, the strengthening and toughening of inherent brittle inorganic materials can also be implemented from the perspectives of materials and cracks. Take the natural nacre as an example, as shown in Fig. 3, its unique organic–inorganic lamellar orderly “brick-mortar” structure (inorganic matter to organism volume ratio = 95 vol %:5 vol %) realizes a wonderful balance between strength and toughness and breaks traditional strength–toughness paradox. The alternating orderly organic layers can work as plastic zones for nacre, which can release the fracture energy through molecular elongation, conducing the rearrangement, slide, and rotation of aragonite nanograins and plastic deformation of ductile organism. Therefore, a high initial fracture toughness is observed for nacre. In addition, the introduced inorganic–organic interfaces are similar as the sacrificial bonds in hydrogels. The relatively weaker interfaces can preferentially bear stress and break to induce tortuous crack propagation and avoid penetrating destruction in inorganic zones, which tremendously increase the fracture energy of nacre [33]. Consequently, high strength and high toughness can be observed simultaneously in nacre. By simulating nacre’s lamellar structure, organic–inorganic composites with high strength and toughness are constructed. Ceramic-based composites have achieved significant breakthroughs in material strength and toughness through advanced methods such as ice template [25,26,34–37], electrophoretic deposition [38], layer-by-layer method [39], and so on. Besides, optimizing the interfacial bonding can further improve the mechanical properties [34].

The strengthening and toughening strategies of nacre are equally adapted to concrete. However, concrete exhibits more significant heterogeneity due to the complexity of multiple hydration products. Thereby, the toughening of concrete should simultaneously consider the multi-scale brittle source to avoid the barrier issue of toughening effect in the scale transfer process. Consequently, in this paper, from the perspectives of material and crack, in the “Toughening Approaches and Principles of Concrete” section, the proposed toughening approaches have been classified according to scales.

## Toughening Approaches and Principles of Concrete

### Molecular structural optimization

**Fig. 4.**
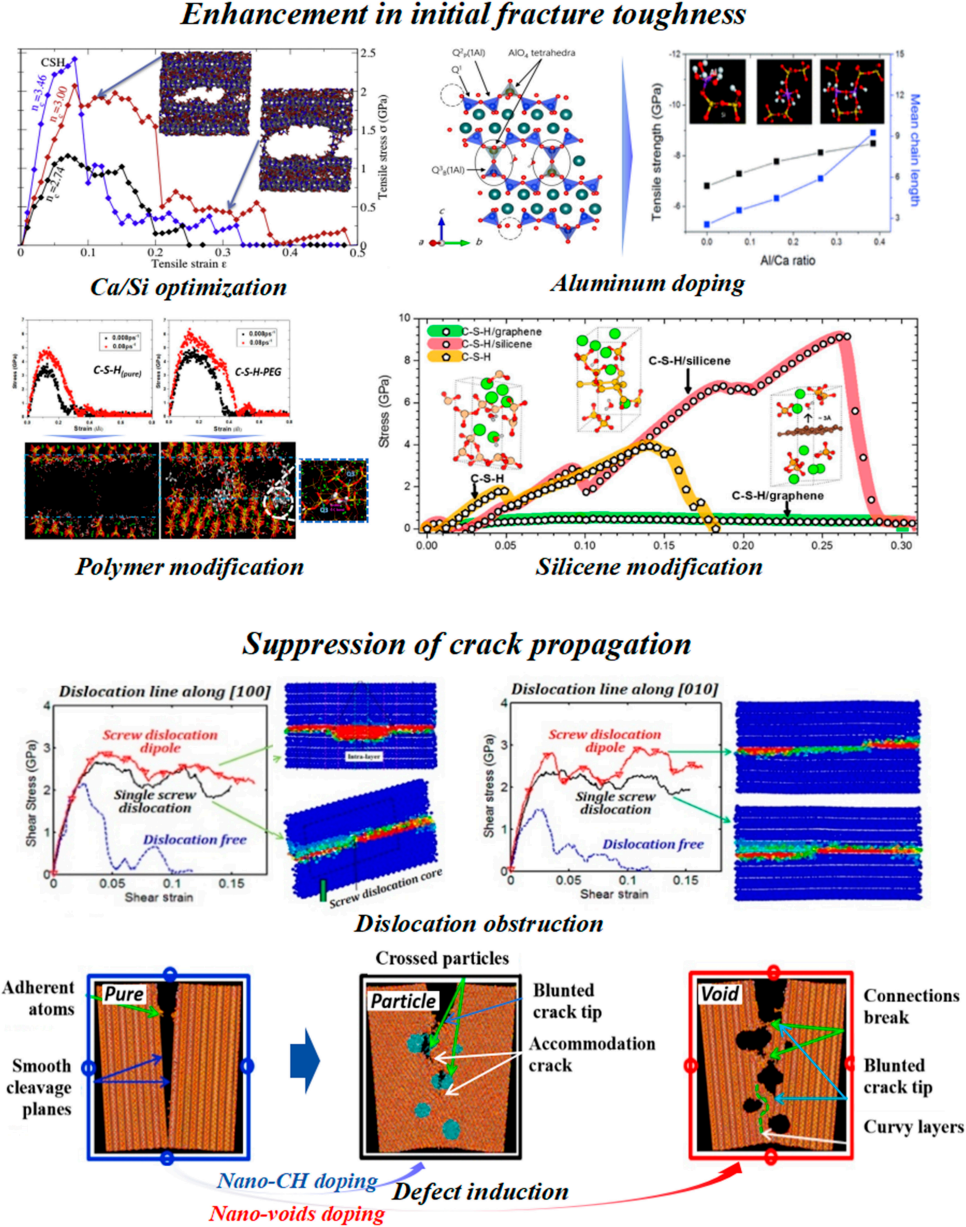
Toughening strategies at the atomic scale. (Top) Enhancement in initial fracture toughness: Ca/Si optimization [21], Al doping [45,46], polymer modification [43], and silicene modification [55]. (Bottom) Suppression of crack propagation: dislocation obstruction [56] and defect induction [57].

C-S-H is the main hydration product of Portland cement-based materials, which is simultaneously the key research subject in nanogenetic engineering of cement-based materials. Since the inconsistent stoichiometry and defective layered molecular structure of C-S-H, the operability of C-S-H brings opportunity and challenge to the optimization in structure and performance [41]. According to the toughening theory summarized in the “Theory of Toughness” section and the present molecular dynamic (MD) simulations and experiments of C-S-H, the strategies for strengthening and toughening of C-S-H at the atomic scale can be divided into 2 parts: molecular structural intrinsic enhancement and suppression of crack propagation, as shown in Fig. 4.

#### Molecular structural intrinsic enhancement

The mechanical properties of C-S-H at the atomic scale are directly related to the fracture rearrangement behavior of the silicate chains. Therefore, the enhancement in initial fracture toughness of C-S-H at the atomic scale mainly involves improving the chaotic state of atomic motions before separation.

Calcium to silicon ratio (Ca/Si), as an essential structural parameter of C-S-H in composition-driven rigidity transition, usually shows opposite relationship with the number of defects in silicate chains (positive correlation) and interlayer spacing (negative correlation) [23]. Another opinion suggests that the defects in C-S-H can provide sites for structural breaking (Si–O or Ca–O bond hydrolysis [42,43]) and regulate the topological constraint environment of atomic motions [21]. Thereby, the fracture process of C-S-H at the atomic scale shows “nanoductility” [21,44]. When Ca/Si value is around 1.5, C-S-H shows an isostatic topological constraint atomic network (n_c_ ≈ 3), which can ensure enough constraints to limit the formation of new fracture surfaces and simultaneously reserve a certain degree of freedom to realize appropriate deformation and alleviate local stress [21]. Therefore, greater deformability at the high tensile stress and better fracture toughness can be obtained at Ca/Si ≈ 1.5.

Additionally, aluminum doping can efficiently increase the mean chain length (MCL) and the polymerization degree through substitution with silicon and bridging effect at the defects in silicate chains. When Ca/Si = 0.95 and Al/Si = 0.20, the MCL of C-A-S-H is 4.86, while the MCL of C-S-H is only 3 at the same Ca/Si [45]. The formation Al–Si 3-dimensional network can stabilize the hydrogen bonding in the interlayer and promote the molecular structural ordering degree [46]. Therefore, higher elastic modulus (21.8% to 39.8%) and tensile strength (30.8% to 100%) of C-S-H have been observed through the incorporation of Al [45].

Besides, the intercalation effect of polymers [polyacrylic acid (PAA), polyethylene glycol (PEG), poly(diallyldimethylammonium chloride) (PDC), poly(methacrylic acid) (PMA), polyvinyl alcohol (PVA), ...] in the interlayer of C-S-H has been proposed through MD simulation [43] and the observed increase of the interlayer spacing in x-ray diffraction (XRD) patterns [47–49]. The nanodefects in silicate chains of C-S-H provide numerous silanol (Si–OH) in the interlayer, which provides plenty of active sites for the condensation reaction and formation of hydrogen bonding between organic polymers and C-S-H. Besides, free calcium ions in the interlayer can also form electrostatic interaction with anionic polymers. Therefore, the bridging effect at the defects in C-S-H has been frequently used for explaining the improved structural connectivity and mechanical properties. Abundant chemical bonding (Ca(COO)_2_ [50,51], hydrogen bonds [52], ...) between functional groups in polymers and C-S-H has been demonstrated through some advanced experiments, such as x-ray photoelectron spectroscopy [50], MD simulation [43,53,54], or time-of-flight secondary ion mass spectrometry (ToF-SIMS) [51]. The formation of Si–O–C bonds between PEG and C-S-H can effectively inhibit the tensile structural fracture caused by the hydrolysis of Si–O–Si or Si–O...Ca bonds [43]. It is worth noting that the improvements mentioned above are all based on the prerequisite of intercalation effect of polymers. However, the differences in bulk modulus and interlayer spacing between high-pressure x-ray diffraction (HP-XRD) experiment and MD simulation for C-S-H/PEG composite reveals the uncertainty about the intercalation effect [54].

Lastly, the 2-dimensional nanomaterial, silicene, shows excellent affinity with C-S-H. It can increase the polymerization degree of C-S-H through Si–O–Si bonds, which is similar as the modification effect of polymers [55]. At the same time, the observed Si–Si bond tilting, pulling, and rotating during the tensile process can efficiently release energy and local stress. Thus, significant improvement in toughness (work of fracture, 118%) and tensile strength (53%) has been obtained. The higher Pugh index value (K/G) implies a transition from brittleness to ductility.

#### Suppression of crack propagation

In addition to the intrinsic enhancement of molecular structure, toughening by optimizing the crack propagation at the atomic scale has also been verified by many researches. The incorporation of screw dislocation into C-S-H can suppress the rapid crack propagation during the whole fracture process by adjusting structural interlaminar gliding path and forming dislocation jog with a climb around the original screw dislocation core [56]. Therefore, higher shear stress and toughness can be obtained. Additionally, nanovoids or nano-CH particles [57] in C-S-H can induce the formation of more tortuous crack path as a result of the crack blunting and occurrence of “accommodation crack”. Optimized crack propagation can expand the energy release during the fracture process and realize a brittle-to-ductile transition of C-S-H.

### Modification of hydration products

**Fig. 5.**
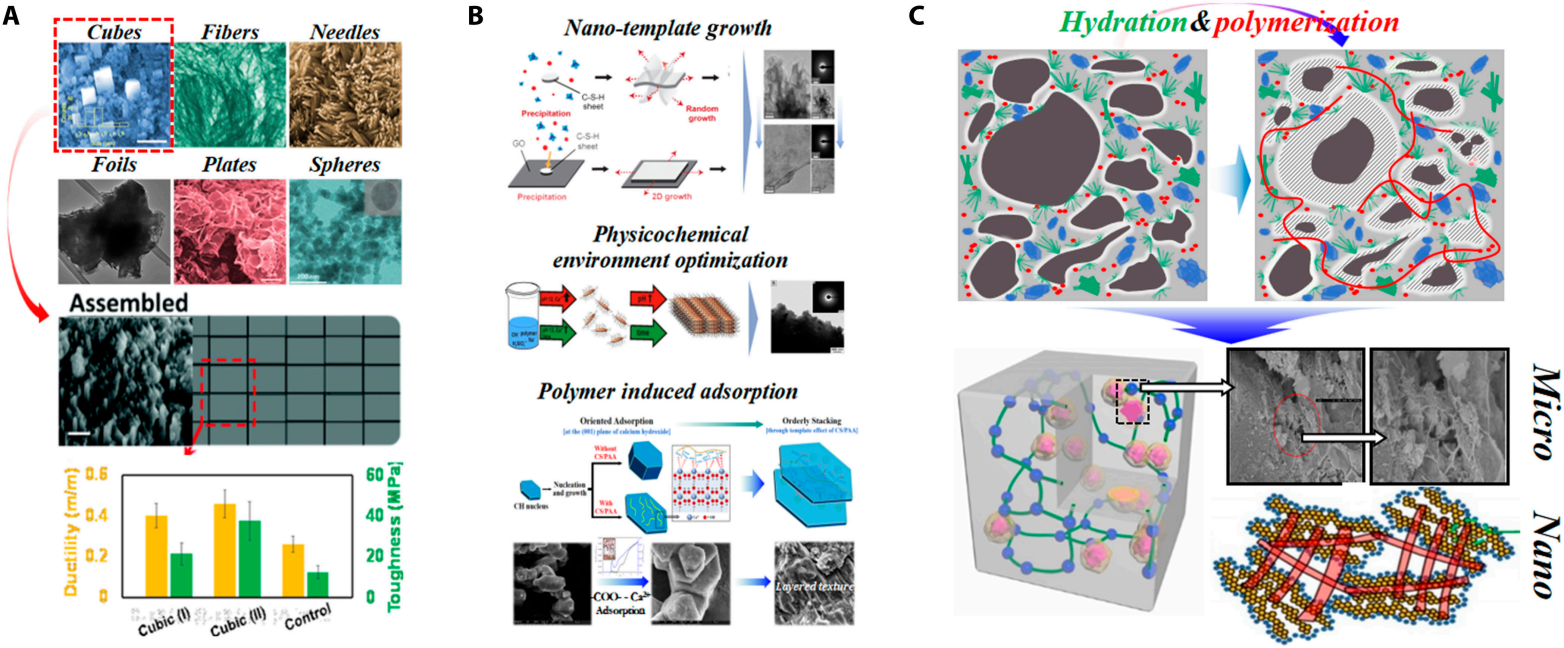
Toughening strategies at the nano- to microscale. (A) Morphological regulation [63]. (B) Orderly stacking [13,64,65]. (C) Interfacial optimization [50,53,66].

At the nano- to microscale, the hydration products of cement-based materials are complex and diverse, bringing significant interfacial problems between hydration products. The packing density and interfacial forces between hydration products initially become the dominant factors in mechanical properties: C-S-H and CH are the main hydration products of cement-based materials. On the one hand, due to the instability of hydrogen bonds and Ca–O bonds at the interfaces, the interfacial fracture of C-S-H/CH composites preferentially occurs, and the fracture toughness is reduced by nearly 50% compared to pure C-S-H or CH [20]. On the other hand, considering the incongruent dissolution of cementitious mineral (C_2_S, C_3_S, C_3_A, and C_4_AF) and diverse stoichiometry of C-S-H, the difference in concentration for various ions within the pore solution can directly affect the cohesion and packing density of C-S-H [58]. Besides, the disorderly stacking and diverse morphology of C-S-H can also cause abundant pores inside matrix during the nucleation and growth process of hydration products. The fracture toughness of high-density C-S-H is (51.7 to 163.1)% higher than that of low-density C-S-H [59,60]. The tensile strength at the nano- to microscale is about (66 to 320) MPa [61], which is (1 to 2) orders of magnitude lower than uniaxial tensile strength of C-S-H at the atomic scale [43]. Single optimization at the atomic scale without considering the structural change at the nano- to microscale will cause unremarkable improvement and even degradation, such as the differences in results between [43] and [54], [45], and [62]. Thus, according to toughening theory summarized in the “Theory of Toughness” section, the toughening strategies at the nano- to microscale should regard hydration products as the main research objects and can be concluded into 3 parts: the morphological regulation to enhance initial fracture toughness, orderly stacking, and interfacial optimization to improve crack propagation (Fig. 5).

#### Morphological regulation

As the main hydration product of cement-based materials, C-S-H generally shows various morphologies because of the diverse chemical stoichiometry and inherent defective Tobermorite structure [42]. C-S-H in cement paste is mostly loosely packed in foil-like, amorphous, or needle-like form [67,68]. By utilizing the hydrogen bonding, hydrophilic–hydrophobic effect, and electrostatic interactions of polymer micelles in aqueous solutions, the self-assembly of C-S-H can be induced by regulating the concentrations of sodium dodecylbenzenesulfonate (SDBS) and hexadecyltrimethylammonium bromide (CTAB). Thereafter, different morphologies of C-S-H nanoparticles can be synthesized, such as the amorphous, spherical, layer-like, and needle-like particles [69]. By combining the self-assembly effect of CTAB and the crystallization induction effect of amorphous calcium carbonate (ACC), cubic C-S-H particles can be obtained in the laboratory [63]. Cubic C-S-H particles are more conducive to dense packing, whose packing density can be increased by about 50% compared with general C-S-H particles. The compressive strength and toughness (fracture work) can be increased by 36% and 300%, respectively.

#### Orderly stacking

Orderly stacking of inorganic materials can effectively reduce the porosity and form a lamellar structure resembling the natural nacre mentioned above to induce more tortuous crack propagation path. Currently, the most efficient regulation measures are generally by using nanotemplates, controlling physiochemical environment, and selective adsorption induced by polymers.

1. Nanotemplates

Appropriate templates can effectively limit the spatial twisting deformation of foil-like C-S-H and regulate the layer-by-layer orderly stacking. There are abundant functional groups on the surface of graphene oxide (GO), which can provide nucleation sites for C-S-H by forming Ca–O bonds and allow C-S-H to homogeneously disperse and spread on the surface of GO. Thereafter, through vacuum filtration, 2-dimensional C-S-H/GO composite nanoparticles can be stacked orderly to obtain lamellar alternating structure, whose tensile strength and strain can reach over 70 MPa and over 3%, respectively, exceeding those of disorderly stacking C-S-H/GO composites [65]. Using the ice-template method to construct lamellar orderly cement/polymer composite can also realize a leap forward improvement in toughness [70]. Nevertheless, currently, the excessive amount of polymer in research seriously reduces the elastic modulus of cement-based materials, which needs further technical improvement.

2. Physiochemical environment

Acrylamide-acrylic acid copolymer (PAAm-co-PAA) can absorb on the surface of C-S-H through hydrogen bonds and Ca^2+^ complexation, which is conducive to the efficient and homogeneous disperse. Then, the orderly stacking of C-S-H can be induced by regulating the pH and the supersaturation of [Ca^2+^] to form “mesocrystalline C-S-H” [64]. The flexural strength of the mesocrystal is about 153 MPa, which is 3 times and (40 to 100) times that of macroscopic defect-free (MDF) cement and traditional Portland concrete. Additionally, due to the incorporation of nanohydroxyapatite into cement, the redistribution of [Ca^2+^] can regulate the nucleation and growth of portlandite (CH). Similarly, the observed lamellar orderly stacking CH can inhibit the penetrating destruction and induce more tortuous crack propagation path [11]. Therefore, the flexural strength is enhanced while maintaining the compressive strength of cement paste simultaneously.

3. Selective adsorption induced by polymers

Because of selective adsorption of Ca^2+^ by polymers or other materials with rich Ca^2+^-sensitivity functional groups, the growth and stacking of CH in the (001) crystal plane direction are conducive, such as cationic polyurethane (PUC) and sulfonated graphene (SGN) [14]. In addition, chitosan modified with polyacrylic acid (CS/PAA) [13] can preferentially selectively adsorb in the (001) crystal direction of CH and induce morphological transition from hexagonal prisms to hexagonal plates. Besides, the templating effect provided by CS/PAA can conduce the orderly stacking of CH to form lamellar CH structure, similar to the “brick-mortar” structure in natural nacre. These orderly structures can work as the local strengthening zones inside cement paste to regulate crack propagation to reduce the fracture energy at the crack tip [71].

#### Interfacial optimization

In addition to the modification effect on the molecular structure of C-S-H, polymers can also optimize the interfacial relationship between hydration products at the nano- to microscale through the chemical bonding (O–Ca–O and O–Na–O bonds [53], Ca(COO)_2_ bonds [50,51], hydrogen bonds [52], ...), manifested as bridging organic membrane [66,72] between products or especially dense C-S-H/organic superstructure with high stiffness and super-low porosity [73]. Therefore, improved interfaces, bridging polymers, and extra deformation from polymers can efficiently enhance toughness. Besides, the synergism of cement hydration and in situ polymerization of organic monomers is beneficial for the formation of stable organic–inorganic 3-dimensional network [50,51,53,74], which can improve the dispersion of polymers in cement paste and reduce the heterogeneity of cement. Compared to directly adding polyacrylamide (PAM), the in situ polymerization of acrylamide monomer (AM) with cement-based materials can effectively improve the strength degradation problem by forming Ca(HOOC)_2_ bonding, achieving a 53% and 26% increase in flexural strength and compressive strength, respectively [51].

### Improvement at the meso-to-macro scale

#### Optimization of ITZ

**Fig. 6.**
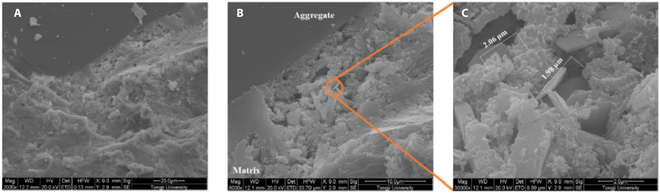
Scanning electron microscopy images of ITZ in concrete [78]. (A) 2,000×. (B) 8,000×. (C) 30,000×.

At the meso-to-macro scale, the average thickness and volume ratio of ITZs between paste and aggregates [75] are about (10 to 50) μm and (20 to 30) vol %, respectively [76,77], as shown in Fig. 6 [78]. The chemical and mineralogical composition of the ITZ are similar to those of the cement paste. However, due to the effects of the edge wall and micro-region water bleeding [79], the average porosity of ITZ is approximately 30% higher than that of the cement matrix [80], which results in a lower packing density, with tensile strength and tensile elastic modulus being 67% and 33% of those of the cement paste, respectively [81,82]. Additionally, the enrichment and preferential orientation growth of CH crystals on the aggregate surface reduce the interfacial bond strength, further exacerbating the overall heterogeneity. This leads to issues such as inconsistencies in performance and noncooperative deformation between the cement paste and aggregates. Consequently, the ITZ is considered a “weak zone” in concrete, prone to localized micro-crack development and harmful ion infiltration. Factors such as ITZ thickness, porosity, and bond strength with the cement matrix are critical in influencing the mechanical properties and durability of concrete. Current researches indicate that the addition of nanomaterials and polymers can effectively reinforce the ITZ in concrete [83].

**Fig. 7.**
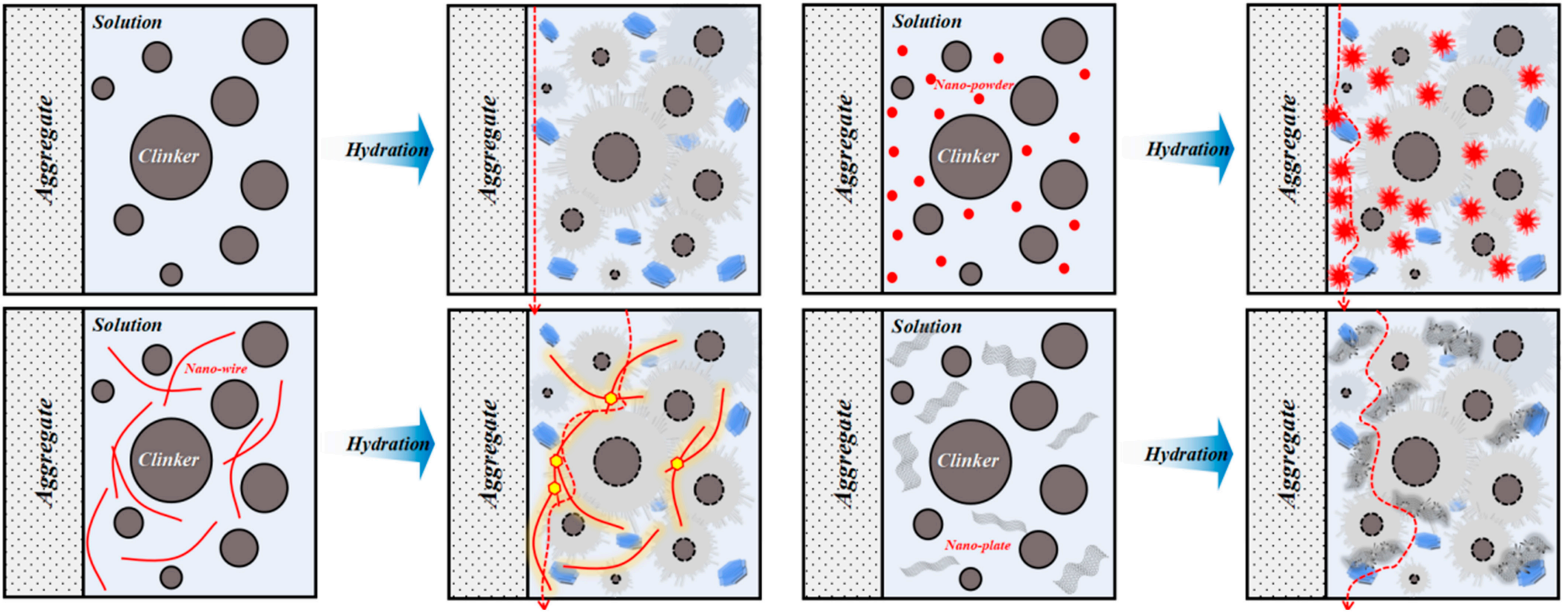
ITZ modification mechanism of nanomaterials [84].

Incorporating nanomaterials directly into concrete or employing them to pretreat aggregates can enhance the microstructure of the ITZ through physical and chemical effects at the nanoscale, thus enhancing the mechanical properties of concrete [75]. This approach has been proved to be a significant method for achieving high-strength and durable concrete, and Fig. 7 illustrates a schematic diagram of the modification mechanism of the concrete ITZ [84].

In detail, as the result of the nucleation effect, pozzolanic effect, filling effect, and bridging effect of nanomaterials [85,86], the morphology and distribution of hydration products within the ITZ undergo changes, including the reduction in the content, size, and orientation of CH crystals, the increase in the content of C-S-H gel, the promotion of orderly growth of C-S-H, and also the decrease in the distance between C-S-H layers [87–89]. Consequently, this leads to a decrease in ITZ porosity and an increase in microhardness. As the strength of the ITZ reaches a certain level, the concrete’s crack failure path shifts from extending along the ITZ to passing through the aggregate [90]. The addition of carbon nanotubes (CNTs) reduces the ITZ thickness by 14% to 30%, and the concrete’s impermeability, which is linearly related to the ITZ thickness, can be improved by approximately 11% to 36% [91]. Furthermore, the incorporation of nano-SiO_2_ can decrease the porosity to ameliorate the microstructure of ITZ, and enhance the 28-day compressive strength, flexural strength, and interface bonding strength of concrete by 30%, 23%, and 43%, respectively [92,93].

The primary techniques for polymer-modified ITZ are as follows: (a) Incorporating polymer molecules directly to fill the micropores and reduce the porosity of ITZ: Silane coupling agents and polyvinyl alcohol enhance the interfacial bonding strength between cement paste and other materials through coupling [94]. When maintaining a polymer–cement ratio below 0.16, the styrene–acrylic emulsion disperses uniformly in the cement paste. Small polymer molecules aggregate to create some membrane in the ITZ of mortar, effectively filling defects within the cement-based material. This process enhances the compactness of the ITZ and improves its mechanical properties. (b) Using polymer to pretreat aggregates: Aggregates treated with asphalt, liquid paraffin, or waste oil can establish a weakened interface. The concrete’s softening curve exhibits a higher critical crack opening displacement, thereby enhancing the fracture toughness of the concrete [95–97]. Nevertheless, due to the premature debonding of the polymer film from the cement paste, the concrete’s strength and modulus experience significant reductions.

In general, at the meso-to-macro scale, the modification of ITZ plays a dominant role in improving the mechanical properties of concrete, and this is mainly achieved by reducing the porosity of ITZ, which is closely related with the water–cement ratio, curing age, and the size and lithology of aggregates. It has been reported that decreasing the water–cement ratio and extending the curing period will lower the porosity of the ITZ and boost the density of hydration products [98–100]. Ultrafine mineral admixtures can diminish the wall effect by leveraging the pozzolanic and filling effects, consequently reducing the porosity of ITZ [101]. Furthermore, variables such as the size, type, saturation, and water absorption characteristics of aggregates will also impact the porosity of the ITZ [102].

#### Bridging effect of fibers

The bridging effect of fibers in concrete after cracking can effectively prevent brittle failure, thereby significantly enhancing the tensile strength and work of fracture of concrete. The report of using fiber to improve the toughness of cement concrete can be traced back to the 1990s [103,104]. As a composite material, the performance of fiber-reinforced concrete is affected by its composition and structural parameters. As the main factor in transmitting stress between fiber and matrix, the study of fiber–matrix interface bonding performance is the basis for understanding and quantifying the toughening and crack resistance behavior of fiber-reinforced concrete, and is also the key to determining the macroscopic tensile behavior of fiber-reinforced concrete, which is usually affected by the concrete matrix properties [105], fiber [106], and interface characteristics [107]. When subjected to external forces, the fiber undergoes 3 consecutive stages: the elastic stage, the elastic bonding–debonding slip stage, and the debonding slip stage as it is pulled out from the concrete matrix. During the initial stage, both the fiber and the concrete matrix contribute to the formation of the initial crack. However, the impact of fibers on suppressing the initial crack is only around 10 to 20%. The subsequent 2 stages determine the fiber’s influence on the toughness of concrete, as it forms the fiber–matrix interface force through chemical bonding force, physical friction force, normal stress, and mechanical anchoring force.

**Fig. 8.**
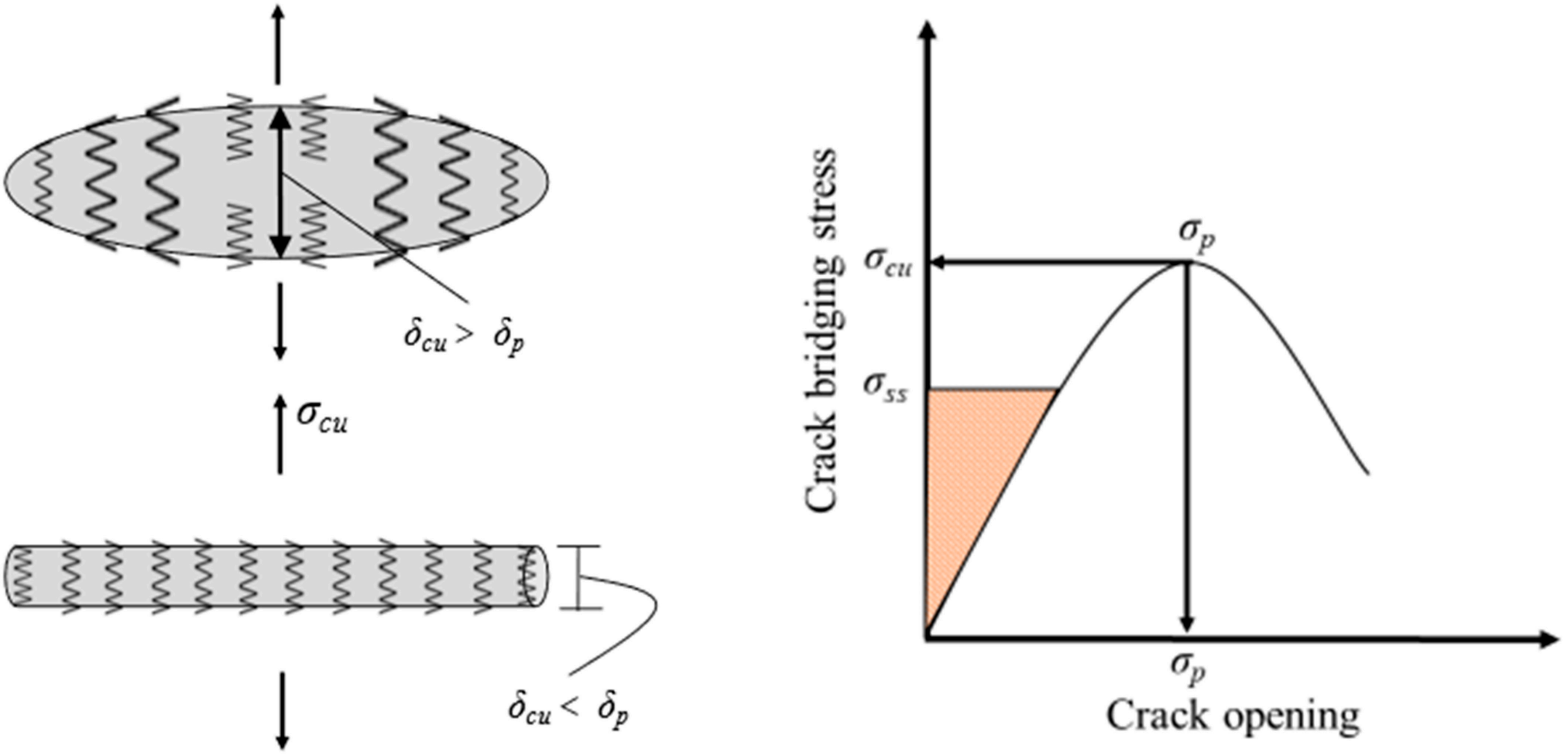
(Left) Unsteady and steady-state crack propagation models. (Right) The relationship between fiber bridging stress and crack opening displacement [110].

The essence of fiber toughening lies in achieving strain hardening and multi-crack cracking, leading to a substantial enhancement in concrete’s fracture toughness and a reduction in crack width. The formation of strain-hardening hinges on the fiber’s ability to suppress non-steady-state crack modes [108], as depicted in Fig. 8 (left). Upon cracking of fiber concrete, numerous fine cracks emerge ahead of the primary crack [109], inducing stress relaxation and exacerbating non-steady-state crack expansion. During non-steady-state crack expansion, the crack is solely influenced by the “bridging fiber” in the middle, resulting in insufficient critical debonding shear stress or maximum bridging stress, thus exhibiting strain-softening behavior. However, by adjusting the fiber–matrix interface properties and the fiber reinforcement factor (*V*_f_*l_f_/*d*_f_), the bridging stress and residual energy of the fiber are augmented, elevating the critical debonding shear stress or maximum bridging stress to a level that fosters a steady-state crack expansion mode, leading to multi-crack cracking and strain-hardening behavior. At this stage, the pivotal control parameter for strain-hardening shifts from fracture energy to the functional relationship between crack expansion and bridging stress.

Related design methods have also been studied by many scholars, among which the J integral method is considered to be one of the most effective methods for designing strain hardening, as shown in Fig. 8 (right). Based on this, the stress criterion and energy criterion for stable crack extension and multi-crack cracking mechanism were proposed and developed, as shown in Eq. 1 [108]. In order to effectively achieve concrete strain hardening, it is usually recommended that σ_pc_/σ_ss_ > 1.2 and *J*_b_/*J*_tip_ > 3 [111]. The higher the redundancy, the greater and more stable the strain hardening of the concrete, until the non-steady-state extension failure of local cracks occurs, and it is often the initial crack.

$$\sigma_{\mathrm{pc}}/\sigma_{\mathrm{cc}}>1;\;\sigma_{\mathrm{cc}}\varepsilon_{\mathrm{cc}}-\int_{0}^{\varepsilon_{\mathrm{cc}}}\sigma \left(\varepsilon \right)d\varepsilon \leq \sigma_{\mathrm{pc}}\varepsilon_{\mathrm{pc}}-\int_{0}^{\varepsilon_{\mathrm{pc}}}\sigma \left(\varepsilon \right)d\varepsilon$$ (1)

## Preparation and Applications of Toughened Concrete

Toughening strategies summarized in the “Toughening Approaches and Principles of Concrete” section are usually implemented in the laboratory rather than in engineering applications. Up to present, considering the feasibility, economy, and functionality required for large-scale preparation in actual large-scale applications, in situ polymerization concrete and fiber-reinforced concrete are the most commonly used toughened concretes.

### Preparation of toughened concrete

#### In situ polymerization of organic monomers

With the development of polymers, new modification methods and technologies are constantly being proposed, especially monomer modification, also known as in situ polymerization of monomers to toughen concrete. The most involved organic monomers are acrylamide (AM), acrylic acid (AA), 2-acrylanmi-do-2-methylpropanesulfonic acid (AMPS), etc. [50,51]. Among them, AM has been widely applied in the cementitious materials because of its high stability in alkaline environment. In situ reaction can improve the toughening effect from the perspective of compatibility, uniformity, and bonding. Due to the simple in situ polymerization of monomers and modification effects, it has been widely studied in the field of ceramic materials. In situ polymerization makes the reinforcing phase evenly dispersed in the matrix, enhances the compactness of the matrix, and improves the fracture toughness of the matrix. The breaking elongation and breaking strength of polymethyl methacrylate (PMMA) obtained by in situ polymerization were increased by 9.6 times and 1.6 times, respectively [112].

Compared with polymer modification, in situ polymerization of monomers has a more significant effect on improving mechanical properties [51]. In situ polymerization can be initiated by adding monomers, initiators, and cross-linking agents during stirring. The mechanical properties of in situ polymerization-modified cement slurry depend on the mix ratio. As the initiator content increases, the flexural and compressive strengths first increase and then decrease [53]. Cross-linking agents can generate chemical bonds to promote the formation of network structures to further improve the toughening effect of in situ polymerization [113]. Sodium acrylate as a monomer can increase the compressive strength by 15% and the flexural strength by 200% after in situ polymerization [53]. As the acrylamide content increases, the polymer tends to aggregate and separate from the cement matrix [51]. Through MD simulations, it is known that the carboxyl functional group of the polymer can generate chemical bonds with C-S-H to enhance the strength [51,53,114]. The in situ polymerization of monomers occurs earlier than the acceleration period of cement hydration [51], so the in situ polymerization of organic monomers will have an impact on the early performance of cement. In situ polymerization accelerates initial setting and retards final setting [53]. The products after the monomer polymerization reaction will adsorb Al ions in the solution and adsorb on the surface of cement particles to inhibit their dissolution, ultimately leading to the delay of cement hydration [115] and the reduction of compressive strength [113]. The interaction between polymers and cement particles can increase the yield stress of cement slurry [116]. It is worth noting that AM has the function of a water-reducing agent [117], so the incorporation of the monomer may increase the fluidity.

Multi-scale collaborative toughening is an efficient method to improve toughness. Recently, the combined impact of in situ polymerization and fibers on enhancing the toughness of cement-based materials, involving nano-whiskers and multi-scale fibers, has emerged as a focal point of research [113,118,119]. Through the regulation of multi-scale hydration microstructures, the toughness of cement-based materials can be greatly enhanced. The in situ polymerized organic matter can produce hydrogen bonds with the fiber surface, which will improve the bonding performance and interface structure between the fiber and the cement matrix, achieve multi-scale collaborative toughening [118], and increase the flexural strength of cement pastes by more than 150% [120]. For concrete, under the synergistic effect of in situ polymerization and nano-whiskers, its 28 d of flexural strength can be increased by 37%, and the fracture energy can be nearly doubled without loss in compressive strength caused by organic polymerization [72].

#### Fiber reinforced concrete

**Fig. 9.**
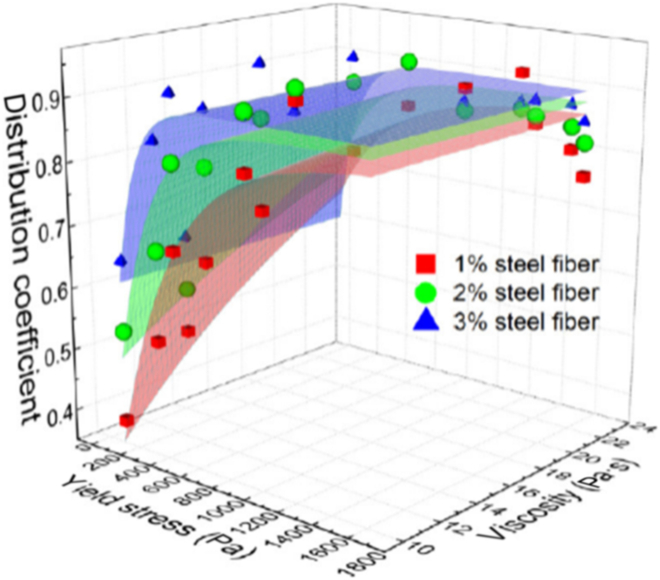
Fiber distribution versus rheological performance [125].

**Fig. 10.**
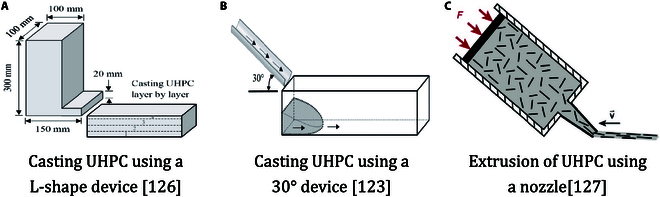
Three optimized casting methods for UHPC. (A) Casting UHPC using an L-shape device [126]. (B) Casting UHPC using a 30° device [123]. (C) Extrusion of UHPC using a nozzle [127].

Numerous studies have shown that the uniformity and orientation of fibers have a significant impact on the toughness of ultra-high performance concrete (UHPC), and their distribution in UHPC is influenced by factors such as fresh mix performance, pouring method, and mold size [121,122]. Reducing yield stress and increasing viscosity can improve the uniformity of fiber distribution [123,124], and the effects of different rheological parameters on fiber distribution are shown in Fig. 9. Increasing the pouring height is detrimental to the uniformity of fiber distribution [122]. For the orientation performance of fibers, both random orientation and directional arrangement have different demand scenarios. Among them, it is difficult to achieve directional arrangement of fibers. Currently, reducing yield stress and increasing viscosity can also enhance fiber orientation. Increasing pouring length [125] and optimizing pouring methods are common measures to optimize fiber orientation arrangement. Figure 10 shows different pouring methods for improving fiber orientation. The use of an L-shaped pouring device can increase fiber orientation by 10% to 40% [126], and the use of a tilted 30° chute can cause shear flow of the mixture. Compared with traditional pouring methods, fiber orientation is increased by 15% to 45% [123], while extrusion pouring (such as 3D printing) can ensure that 70% of the fibers are arranged along the pouring direction [127]. In addition, by applying an external electromagnetic field, steel fibers can be aligned along the direction of electromagnetic force, and the fiber orientation can be improved by about 80% compared to the absence of an electromagnetic field [128].

The commonly used fibers for concrete toughening can be divided into 3 categories: synthetic fibers, mineral fibers, and metal fibers. Their properties can be seen in Table 1 [110].

**Table 1.** Fibers used for toughening and crack prevention of concrete [110]

Types of fibers		Specific weight (kg/m^3^)	Flexural strength (MPa)	Elastic modulus (GPa)	Fracture strain (%)	Contact angle (^o^)
Synthetic fibers	PVA	1.30	1,600	42.8	6	60.6
	PE	0.97	1,950–3,000	39–100	3.1–8.0	78–96
	PP	0.91–0.97	850–928	2.7–9.0	7.3–30	102.1
Metallic fibers	Steel	7.80	350–3,000	210	2–4	81.4–87.2
Mineral fibers	Carbon	1.57–1.80	525–4,660	33–268	0.8–2.4	29–84.7
	Basalt	2.60–2.80	2,230–4,840	85.8–89.0	2.8–3.1	68
	Glass	2.60	2,000–4,000	70–80	2.0–3.5	78.9

Mineral fiber can significantly improve the tensile strength of fiber reinforced concrete due to its high tensile strength and elastic modulus. However, due to its low deformation performance caused by poor shear resistance, mineral fiber cannot effectively improve the ductility of concrete, resulting in lower fracture energy. At the same time, mineral fibers usually have poor interfacial physical friction. Compared with other fibers, they are not suitable for the preparation of strain-hardening concrete and the control of crack width. They are often made into fiber-toughened polymers and used to toughen concrete structures. It can even be used instead of steel bars.

Synthetic fibers and steel fibers are generally used in concrete as short-cut fibers to improve tensile properties and fracture toughness. However, compared with steel fibers, synthetic fibers usually have a smaller elastic modulus and a larger elongation at break. To satisfy the strain-hardening requirement, synthetic fibers are usually used in engineered cementitious composite (ECC), while steel fibers are often used in UHPC materials. As shown in Table 2, the volume content of synthetic fibers in ECC is generally (0.75 to 2.5)%, its tensile strength is (3 to 5) MPa, and the tensile strain can reach (3 to 10)%. The ECC obtains a very high fracture energy through the larger tensile strain. It should be noted that due to the larger elongation at break of synthetic fibers, the appearance of new cracks during the stretching process will produce larger bearing stress fluctuations. Based on outstanding dispersibility and suitable interfacial bonding, PVA fiber is the most commonly used fiber for preparing ECC, but due to its strong hydrophilicity, its chemical bonding force with the matrix is often too large and may even exceed the physical friction force, which is not conducive to the satisfaction of the strain-hardening energy criterion. In addition, as hydration proceeds, the chemical bonding between PVA fiber and the matrix will further increase, reducing the ultimate strain of fiber concrete. Therefore, the PVA fiber surface is coated to reduce the chemical bonding strength and achieve improved ductility of concrete. Polypropylene (PP) fiber has a lower elastic modulus and higher ductility (self-deformation) so that the prepared ECC usually exhibits weaker tensile strength but better ductility. It is also considered to be an important fiber to replace PVA fiber and optimize the cost of ECC. However, compared with PVA fiber, PP fiber will adopt surface treatment to improve its chemical bonding. Since polyethylene (PE) has high tensile strength, elastic modulus, and ductility, the ECC prepared by PE usually exhibits high tensile strength and ultimate strain. Even the tensile strength of ECC prepared by high-strength PE fiber can exceed 20 MPa, but it should be noted that compared with PVA fiber, its ability to suppress crack width is poor. The volume content of steel fiber in UHPC is generally (1.5 to 3.0)% [129]. Metal fibers can significantly improve the tensile strength of concrete due to their extremely high tensile strength and elastic modulus, but due to their small elongation at break, the strain of fiber concrete is usually less than 1.5%. Compared with synthetic fibers, the interfacial force between metal fibers and the matrix is significantly improved, with chemical bonding stress reaching (4 to 100) MPa and physical friction reaching (1 to 10) MPa [3]. As the strength of the concrete matrix increases, the interfacial bonding strength is further improved. For example, the interfacial bonding strength of fibers and matrix can reach more than 20 times that of ordinary and high-strength concrete [130]. In addition, compared with synthetic fibers, the plasticity of metal fiber shape can further improve its interfacial bonding strength, thereby obtaining better tensile fracture energy and crack control ability. Twisted fibers are the best, followed by end hook fibers, and straight fibers.

**Table 2.** Influence of fiber type on tensile properties of concrete [129]

Types of fibers	Dosage (%)	Interfacial force (MPa)		Initial tensile strength (MPa)	Ultimate tensile strength (MPa)	Ultimate strain (%)	Crack width (μm)	Crack spacing (mm)	Fracture energy (kJ/m^2^)
		Chemical bonding force	Physical friction force						
PVA	0.75–2.5	2.50–5.70	1.30–1.90	2.6–4.0	3.9–5.0	1.4–4.6	42–71	1.8–11.5	5.4–6.5
PP		/	0.20–0.45	1.4–3.2	2.3–4.3	0.8–3.9	63–258	2.4–15.5	/
(HD)PE		/	0.40–1.02	4.4–8.3	6.6–11.9	3.4–9.6	50–150	1.3–8.0	5.4–16.0
SF	1.5–3.0	/	8.7–14.2	6.5–12.5	11.3–17.8	0.17–0.45	10–30	3.2–4.6	22.1–25.6
HF		/	42.4–43.0	5.7–11.0	11.7–19.3	0.42–0.48		4.6–5.8	21.8–30.2
TF		/	24.8–29.0	5.8–13.3	11.6–19.6	0.47–0.61		3.0–4.0	19.5–25.3

**Fig. 11.**
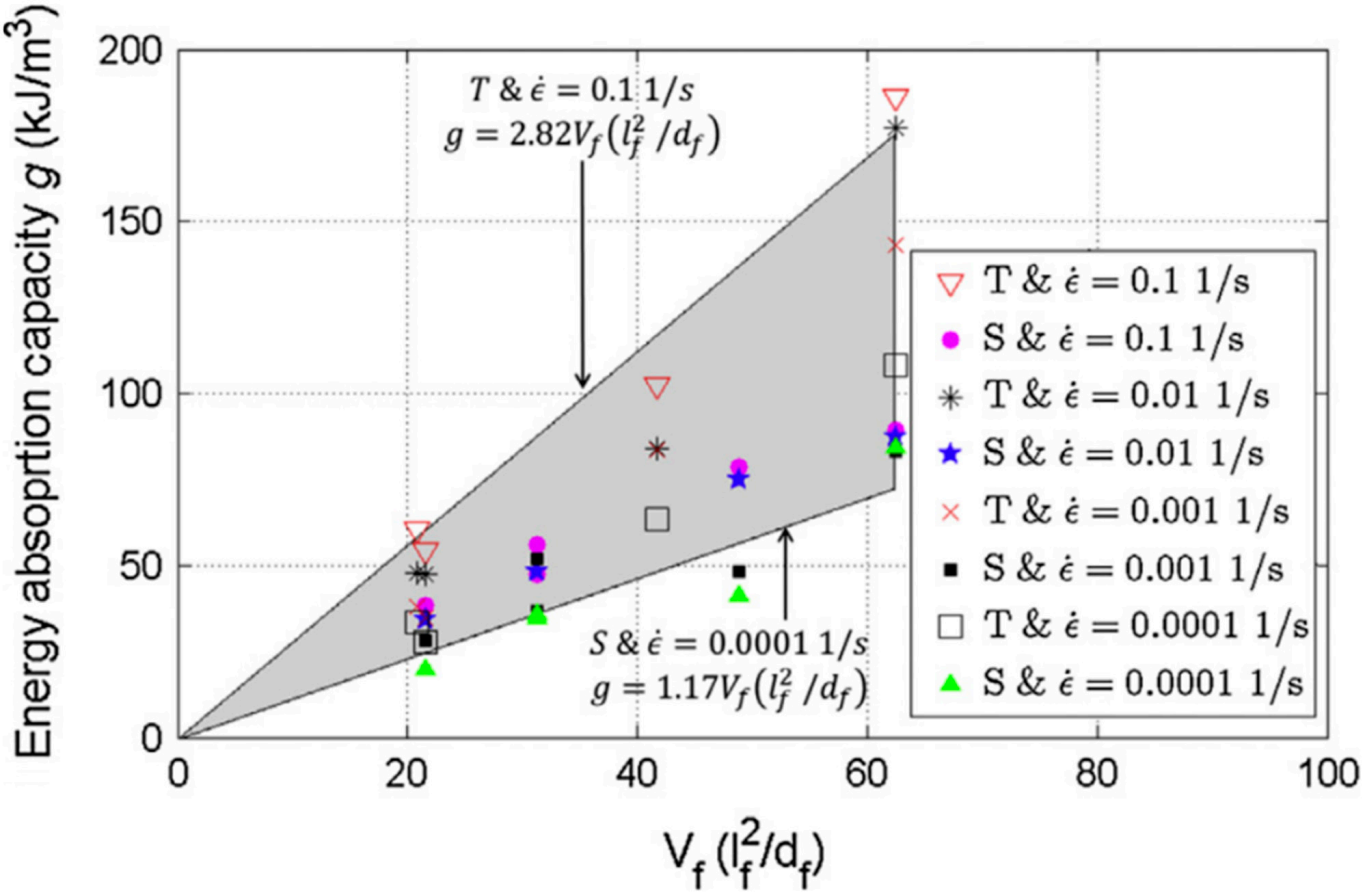
Effect of fiber reinforcement factor on UHPC fracture energy [132].

However, it should be noted that complex special-shaped fibers cannot obtain better stretching than straight fibers in the manufacturing process, thereby obtaining high tensile strength. It is easy to cause the special-shaped fibers to break during the pull-out process, resulting in relatively poor tensile fracture energy [131]. In addition, increasing the fiber reinforcement factor and fiber mixing are 2 important ways to improve the tensile strength and fracture energy of fiber concrete. As shown in Fig. 11 [132], the tensile strength and ultimate strain of concrete are linearly increasing with the fiber reinforcement factor, which can significantly improve the fracture energy of concrete. As shown in Table 3 [110], this is inseparable from the fiber bridging stress and crack propagation function.

**Table 3.** Influence of hybrid fiber on tensile properties of concrete [110]

Hybrid fibers	Initial tensile strength (MPa)	Ultimate tensile strength (MPa)	Ultimate strain (%)	Crack width (μm)	Crack spacing (mm)
SF2.5	7.40	14.2	0.24	7.0	8.02
SF1.5 + Macro SF1.0	9.88	13.21	0.36	3.0	6.66
SF1.5 + Macro HF1.0	10.51	13.84	0.56	6.4	5.15
SF1.5 + Macro TF1.0	10.69	18.56	0.63	4.7	3.80
SF2.5	4.51	6.06	0.19	/	/
SF1.0 + PVA1.5	4.37	5.74	0.48	32.0	/
SF0.5-PVA2.0	3.95	5.13	5.08	57.0	/
PE1.5 + SF0.5	6.50	10.89	4.42	111.8	4.89
PE1.5 + SF1.0	8.49	11.67	5.11	106.4	3.59
PE1.5 + SF1.5	10.05	11.17	3.13	99.8	2.80

Under the same dosage of fibers, the increase of the long fiber content can not only reduce the concrete crack spacing by 52% and increase the ultimate strain by 160% but also increase the tensile strength by 30% and refine the crack width by 33%. With the increase of the synthetic fiber content, the ultimate strain of concrete can be increased by 25.7 times, but fibers will reduce the ultimate tensile strength by 16%. At a fixed synthetic fiber content, with the increase of steel fiber content, the tensile strength of concrete will gradually increase, but the ultimate strain will first increase and then decrease. This depends on the critical content of the steel fiber when it plays a leading role in the crack healing process.

### Practical engineering application

**Fig. 12.**
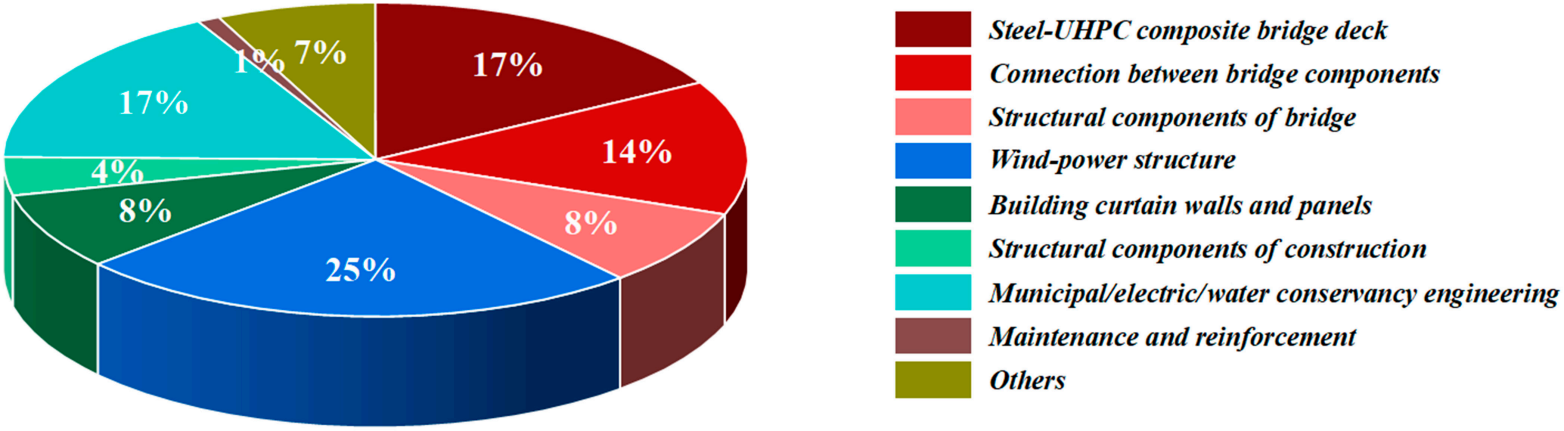
China UHPC application distribution in 2023.

Facing the increasing toughness requirements of concrete, with the technical direction of improving the brittleness of the cement matrix and using fiber toughening, UHPC, as the typical representative toughened concrete, has been fast developed and widely applied. UHPC, which is composed of ultra-high-strength mortar and high-modulus fiber with higher compressive strength, can significantly improve the interfacial bonding strength and crack bridging effect of the fiber [133–135], and its compressive strength can reach (120 to 200) MPa, elastic modulus (45 to 60) GPa, and tensile strength above 10 MPa [129]. Its application scenarios have been widely distributed in bridges, construction, municipal administration, wind power, and other fields. Statistics in 2023 show that China’s UHPC consumption exceeds 146,500 m^3^, and the main application distribution is shown in Fig. 12. Among them, the largest engineering application field of UHPC in China is still bridges, accounting for 39%, mainly steel–UHPC composite bridge decks and bridge component connections (wet joints), followed by wind power structures (prefabricated towers) that have developed rapidly in recent years (Bobbin sheets), accounting for 25%; municipal, electric power, and water conservancy projects, accounting for 17%; construction applications that are mainly prefabricated curtain walls, wall panels, and structural components, accounting for about 12%; maintenance and reinforcement consumption, accounting for about 1%.

**Fig. 13.**
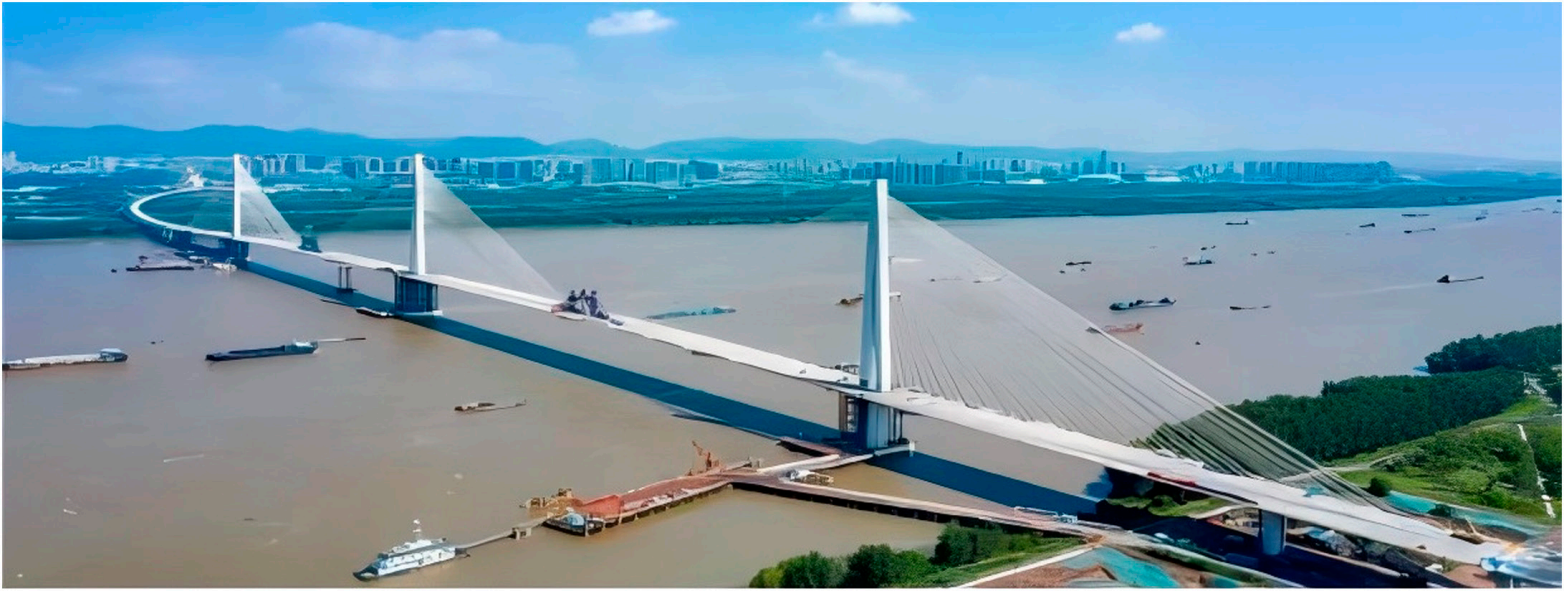
The Fifth Nanjing Yangtze River Bridge.

Due to its ultra-high mechanical properties and excellent durability, UHPC can significantly reduce structural size and improve structural toughness. It is a breakthrough material for achieving lightweight and high-performance bridge structures. The Fifth Nanjing Yangtze River Bridge is a steel–concrete composite structure cable-stayed bridge (Fig. 13). The main bridge span is 1,796 m. The main beam top plate uses a UHPC bridge deck with a thickness of 17 cm. The UHPC used has the characteristics of high flexural tensile strength (first crack strength greater than 10 MPa), high elastic modulus (elastic modulus greater than 54 GPa), and low shrinkage (shrinkage deformation less than 300 × 10^−6^) [129]. The tensile strain of coarse aggregate UHPC is 10 times that of ordinary concrete, and it has excellent crack restraint ability, and the performance indicators are shown in Table 4 [136]. Compared with traditional steel–ordinary concrete composite beams, the use of UHPC can reduce the thickness of the roof from 25 cm to 17 cm, increase the crack resistance of the main beam by 4 times, and reduce creep stress by 70%. Compared with cable-stayed bridges of the same span, the amount of steel used in the main beam is reduced from 400 kg/m^2^ to 311 kg/m^2^, reducing the weight of the main beam by more than ^1^/_3_, and increasing the span capacity of the steel–concrete composite beam cable-stayed bridge to the kilometer level. At the same time, due to the lightweight of the main beam, compared with the traditional steel–ordinary concrete composite beam, the number of beam segments has been reduced from 29 to 20, the amount of steel bars has been reduced by more than 2,000 tons, the overall concrete amount has been reduced by more than 22,000 m^3^, and carbon dioxide emissions have been reduced by 25,545 tons.

**Table 4.** The performance indicators of the Fifth Nanjing Yangtze River Bridge [136]. The shrinkage deformation test age is 90 d of standard curing, and the rest are 28 d of standard curing.

Compressive strength/MPa	Elastic modulus/GPa	Initial cracking strength/MPa	Ultimate flexural strength/MPa	Shrinkage deformation/10^−6^
165.0	56.4	11.5	23.5	216.0

**Fig. 14.**
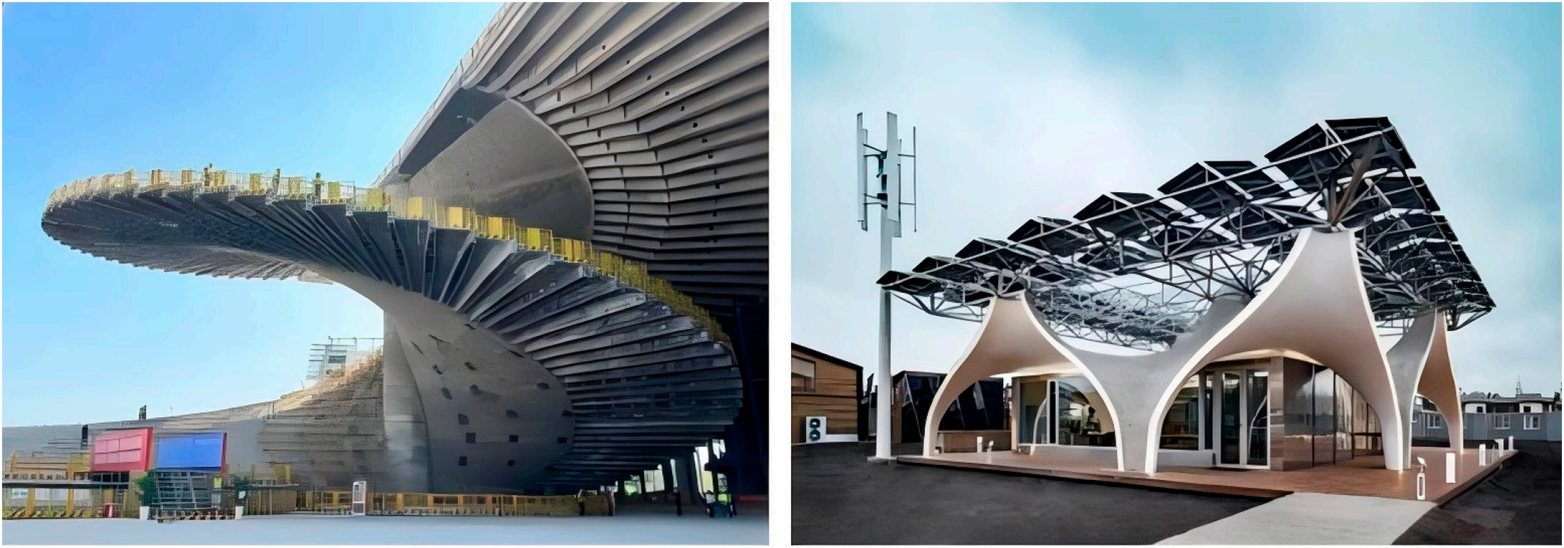
Shanghai Grand Opera House (left) and Solar Ark 3.0 (right).

With its excellent plasticity, mechanical properties, and durability, UHPC provides designers with more creative and practical options, which greatly promotes the sustainable development and aesthetic design of lightweight structures in buildings. As the world’s longest cantilever beam application building (Fig. 14, left), the Shanghai Grand Opera House has a typical asymmetric structure, and its cantilever beam length reaches 15 m. By using nanomaterials to enhance the ITZs between fibers and concrete matrix, the toughening effect of multi-scale fiber by incorporating nano-whiskers–organic fibers–steel fibers can be significantly improved in UHPC. The compressive strength of the UHPC used exceeds 165 MPa, and the tensile strength is greater than 14 MPa. The specific performance indicators are shown in Table 5. The span-to-height ratio of the prepared cantilever beam is 21, which is far higher than the span-to-height ratio of 4 to 6 of conventional cantilever beams. It replaces steel structures, greatly saves maintenance costs, and expands the system innovation of building structures. In addition, since UHPC can achieve the unity of simplified internal structure and complex external design of components, it has broad application prospects in the field of green buildings. By using the injection molding process, a hyperbolic splicing unit with no structural reinforcement and a projected area of 15 m^2^ was prefabricated to build a carbon-neutral zero-energy demonstration building—“Solar Ark 3.0” (Fig. 14, right), which saves 67% of concrete and promotes the development of integrated building structure insulation and decoration.

**Table 5.** The performance indicators of UHPC used in the Shanghai Grand Opera House (data come from the material technical indicators of the project). Standard curing for 28 d.

Compressive strength/MPa	Elastic modulus/GPa	Ultimate flexural strength/MPa	Tensile strength/MPa
166.5	61.3	25.5	14.5

## Prospective Work in Toughing Concrete

This paper focuses on the multi-phase, multi-scale, and heterogeneous characteristics of modern concrete, expounds the essential characteristics of multi-scale brittleness of concrete, and reviews the theory, preparation methods, and engineering applications of multi-scale toughening of concrete. However, the decomposition of raw materials and coal combustion in the production process of silicate cement will emit a large amount of CO_2_, resulting in high carbon emissions in the civil engineering industry, which is difficult to meet the implementation of the national carbon strategy. Fly ash and other mineral admixtures as supplementary cementitious materials for concrete provide a guarantee for low carbon and high performance of concrete. However, raw materials such as supplementary cementitious materials are still unsustainable. With the transformation of the national energy structure, traditional mineral admixtures based on coal-fired power energy will be greatly reduced. In recent years, increasing the proportion of silicon–aluminum phases in cement by blending active aluminum-rich phases such as calcined clay or directly adding calcium carbonate to form a new generation of aluminosilicate cementitious material system can contribute to reduce carbon emissions. Based on this concept, the following aspects need to be focused on in the new aluminosilicate cementitious system:

1. How to accurately understand the problem of multi-phase and multi-interface in aluminosilicate cementitious system?

The addition of aluminum phase in the aluminosilicate cementitious system changes the original hydration product composition. C-A-S-H gel phase, AFt, and AFm crystal forms are generated from new system with multi-phase characteristics of crystal phase–amorphous phase (gel phase)–inert phase. Although the substitution of aluminum can increase the polymerization degree of the silicon chain of C-A-S-H at the nano-micro scale and optimize the pore structure, there is still controversy about the mechanical property enhancement effect brought by aluminum doping. More importantly, the addition of aluminum phase can form more hydration product phases, leading to significant interfacial problems between products and exacerbating the problem of weak interfaces in low-carbon cementitious material systems, resulting in poor deformation performance and prominent brittleness.

2. Is the traditional high-strength and toughness technology of concrete still applicable to the aluminosilicate cementitious system?

The early hydration reaction activity of the aluminosilicate cementitious system is low, the alkalinity and CH content are low, and the main hydration product is C-A-S-H, which is a polycrystalline phase. The characteristics of polycrystalline phase introduce more defects and interface problems. The adaptability of traditional toughening theory to new cementitious material systems needs further discussion. It is worth noting that the doping of Al^3+^ in the aluminum-rich phase provides new opportunities and challenges for the bonding between the product and the polymers or nanomaterial. The construction of the 3-dimensional network structure and the regulation of the microstructure need further study.

## Conclusion

The paper thoroughly investigated the multi-scale brittleness characteristics of concrete materials, encompassing nano, micro, meso, and macro multi-scale perspectives, while delving into the theory of material toughness. It introduces effective multi-scale toughening strategies for concrete and outlines the practical application of high-strength concrete in engineering, offering theoretical backing and technical direction for enhancing concrete toughness and crack resistance. The main conclusions are as follows:

1. The multi-scale structural characteristics of concrete mainly manifested the covalent–ionic bonding at the atomic scale, disordered stacking of hydration products at the nano- to microscale, pore defects at diverse scales, and weak ITZs. When subjected to external forces, uncoordinated deformation induces cracks, which subsequently propagate in a noncontinuous manner, ultimately resulting in fracture. This exemplifies the inherent brittleness of concrete.
2. Adjusting the Ca/Si ratio, incorporating Al doping, and integrating hybrid flexible polymers represent effective strategies for enhancing the toughness of the C-S-H gel unit. Optimizing the morphology of hydration products, controlling the orderly arrangement of these products, and establishing organic plastic zones are crucial for reducing matrix porosity, reinforcing interface deformation, and deflecting cracks. These methods are vital for achieving toughness of concrete.
3. Incorporating fiber materials and organic polymers can effectively bridge and refine cracks, facilitate controlled crack propagation in concrete materials, and greatly enhance its ductility and fracture energy. The higher the fiber strength and modulus, the better the effect of improving concrete tensile strength and thinning cracks. Besides, the higher the fiber elongation at break, the stronger the ability to improve the ductility of concrete.
4. In situ polymerization and fiber toughening are currently practical and efficient methods for toughening concrete, which can increase the tensile strength of the matrix by 30%, as well as the fracture energy by about one time, achieving an ultimate tensile strength of 20 MPa and a tensile strain greater than 0.6%. UHPC has become the most prevalent form of toughened concrete, driving innovations in lightweight design, disease resistance, and the sustainable development of engineering structures like bridges and buildings.
